# Whole genome sequencing analysis of multiple *Salmonella* serovars provides insights into phylogenetic relatedness, antimicrobial resistance, and virulence markers across humans, food animals and agriculture environmental sources

**DOI:** 10.1186/s12864-018-5137-4

**Published:** 2018-11-06

**Authors:** Suchawan Pornsukarom, Arnoud H M van Vliet, Siddhartha Thakur

**Affiliations:** 1 Faculty of Veterinary Medicine, Rajamangala University of Technology Tawan-ok, Chonburi, Thailand; 2 School of Veterinary Medicine, Faculty of Health and Medical Sciences, University of Surrey, Surrey, UK; 3 Department of Population Health and Pathobiology, College of Veterinary Medicine, North Carolina State University, Raleigh, NC USA; 4 Comparative Medicine Institute, North Carolina State University, Raleigh, NC USA

**Keywords:** *Salmonella*, WGS, Core genome, SNP, FFP, Antimicrobial resistance, Virulence gene, Plasmid, Human, Swine, Poultry, Environment

## Abstract

**Background:**

*Salmonella enterica* is a significant foodborne pathogen, which can be transmitted via several distinct routes, and reports on acquisition of antimicrobial resistance (AMR) are increasing. To better understand the association between human *Salmonella* clinical isolates and the potential environmental/animal reservoirs, whole genome sequencing (WGS) was used to investigate the epidemiology and AMR patterns within *Salmonella* isolates from two adjacent US states.

**Results:**

WGS data of 200 *S. enterica* isolates recovered from human (*n* = 44), swine (*n* = 32), poultry (*n* = 22), and farm environment (*n* = 102) were used for in silico prediction of serovar, distribution of virulence genes, and phylogenetically clustered using core genome single nucleotide polymorphism (SNP) and feature frequency profiling (FFP). Furthermore, AMR was studied both by genotypic prediction using five curated AMR databases, and compared to phenotypic AMR using broth microdilution. Core genome SNP-based and FFP-based phylogenetic trees showed consistent clustering of isolates into the respective serovars, and suggested clustering of isolates based on the source of isolation. The overall correlation of phenotypic and genotypic AMR was 87.61% and 97.13% for sensitivity and specificity, respectively. AMR and virulence genes clustered with the *Salmonella* serovars, while there were also associations between the presence of virulence genes in both animal/environmental isolates and human clinical samples.

**Conclusions:**

WGS is a helpful tool for *Salmonella* phylogenetic analysis, AMR and virulence gene predictions. The clinical isolates clustered closely with animal and environmental isolates, suggesting that animals and environment are potential sources for dissemination of AMR and virulence genes between *Salmonella* serovars.

**Electronic supplementary material:**

The online version of this article (10.1186/s12864-018-5137-4) contains supplementary material, which is available to authorized users.

## Background

Infection with antimicrobial resistant *Salmonella* in humans and animals is a global threat that has caught the public attention worldwide [1–3]. Human foodborne salmonellosis causes an estimated 100,000 domestic cases and 40 deaths annually in the United States [1]. The U.S. Department of Health and Human Services reported an increase in *Salmonella* infections from 13.6 to 16.4 cases per 100,000 population, which represented a 17.1% increase from 1997 to 2011 [4]. In the European Union, *Salmonella*-infected gastroenteritis was the second most frequently reported foodborne illness with 91,408 clinical cases reported by thirty EU/EEA countries, and a confirmed case rate of 25.4 cases per 100,000 population in 2014 compared to 21.4 cases per 100,000 population in 2013, which represented a 19% increase in the notification rate [3].

Inappropriate use of antimicrobials in livestock production and the association to resistant *Salmonella* infection in humans are a growing concern to public health agencies, and have led to the rise of new multidrug resistant (MDR) bacteria and transferable genetic loci, such as colistin resistance mediated by the MCR-1 gene [5, 6]. Given the ever-growing requirement to maintain the efficacy of antimicrobials as well as decrease the emergence of antimicrobial resistance in human infections, the antimicrobial use in veterinary and agricultural practices is being extensively re-evaluated [7–9]. Humans and animals are linked to each other through the environmental reservoirs which have long been implicated as a source of *Salmonella* and antimicrobial resistance found in human and animals [8–10]. The selection pressure on *Salmonella* is created by antimicrobial use in human health and food animal production leading to development and potential spread of antimicrobial resistance [8–11]. Our previous studies reported the persistence and dissemination of multiple resistant *Salmonella* serovars along with their determinants in the environment of commercial swine operation due to the manure application on land [12, 13].

Multiple *Salmonella* serovars, including Agona, Anatum, Derby, Heidelberg, Infantis, Kentucky, Muenchen, Newport, Schwarzengrund, and Typhimurium are commonly detected in food animals, food products, and agricultural environments, and are associated with resistant *Salmonella* infections in humans [14–17]. The Centers for Disease Control and Prevention (CDC) reported that the incidence of human *Salmonella* infections caused by monophasic 4,[5],12:i:-, which is in the top 4 of the most frequently reported *Salmonella* serovars, continue to rise while the incidence of the other serovars is decreasing [16]. The increase in the incidence of this serovar in human cases is paralleled by a similar increase in swine and environmental detection of this serovar variant [12, 13, 18]. However, there are gaps that still exist in our understanding of the temporal and spatial connection of resistant *Salmonella* transmission within humans, animals, and the environment sources.

A number of studies have used the classical molecular typing methods such as pulsed-field gel electrophoresis (PFGE), multilocus sequence-based typing (MLST), and multilocus variable-number tandem repeat analysis (MLVA) to assess the relatedness and the subsequent transmission of antimicrobial resistant (AMR) *Salmonella* in human, animals, and environment [19–21]. However, the limitation of these methods lies in insufficient discriminatory power to separate closely related *Salmonella* isolates in outbreak investigations and to differentiate between the intra-serovar isolates from different hosts [20–22]. The use of whole genome sequencing (WGS) has had a major impact on the study of the molecular epidemiology of AMR bacterial pathogens associated and transmitted between human, animal and environmental sources. A WGS study in Denmark reported that SNP, pan-genome, k-mer and nucleotide difference trees were superior to the classical typing method and evaluated the association of the isolates to specific outbreaks of *S.* Typhimurium [23]. Additionally, WGS has been used to identify known AMR determinants among strains of *Escherichia coli* and *Salmonella* [24, 25]. The objectives of this study were to use WGS to analyze multiple *Salmonella* serovars isolated from human, food-animals and environments in the two states of the US and to clarify the epidemiological transmission of AMR *Salmonella* within these studied populations. In addition, the capability of WGS to predict antimicrobial resistance and virulence genes in antimicrobial resistant *Salmonella* retrieved from different sources was evaluated.

## Results

### *Salmonella* serotyping based on WGS

The 200 *Salmonella* sequences in this study selected from human clinical cases, swine, poultry, and environmental samples were serotyped using the SISTR platform for confirmation [26], and showed a high level of serotype diversity (Table 1). The predominant serovars which originated from multiple sources were Derby (*n* = 21), Kentucky (*n* = 5), Johannesburg (*n* = 9), Mbandaka (*n* = 12), Rissen (*n* = 14), Schwarzengrund (*n* = 22), Senftenberg (*n* = 12), Typhimurium (*n* = 39), and 4,[5],12:i:- (*n* = 8).

**Table 1:** Number of *Salmonella* isolates (*n* = 200) from human, animal, and environment by serotype sequenced for comparison

*Salmonella* serotype (*n*)	Source of isolate				
	Human (*n* = 44)	Swine (*n* = 32)	Poultry (*n* = 22)	Environment (*n* = 102)	Total (*n* = 200)
Altona				11	11
Anatum				1	1
Braenderup				1	1
Chester	1				1
Derby	9	7		5	21
Enteritidis	1				1
Heidelburg	1			1	2
Kentucky			4	1	5
Johannesburg		4		5	9
Mbandaka	1	3	3	5	12
Muenchen	9				9
Muenster				16	16
Ouakam		1			1
Rissen		6		8	14
Schwarzengrund	7		7	8	22
Senftenberg		6	3	3	12
Typhimurium	15	5	1	18	39
4,[5],12:i:-			4	4	8
Uganda				4	4
Worthington				11	11

### Comparison of FFP with SNP-based phylogeny of *Salmonella* isolates

**Fig. 1:**
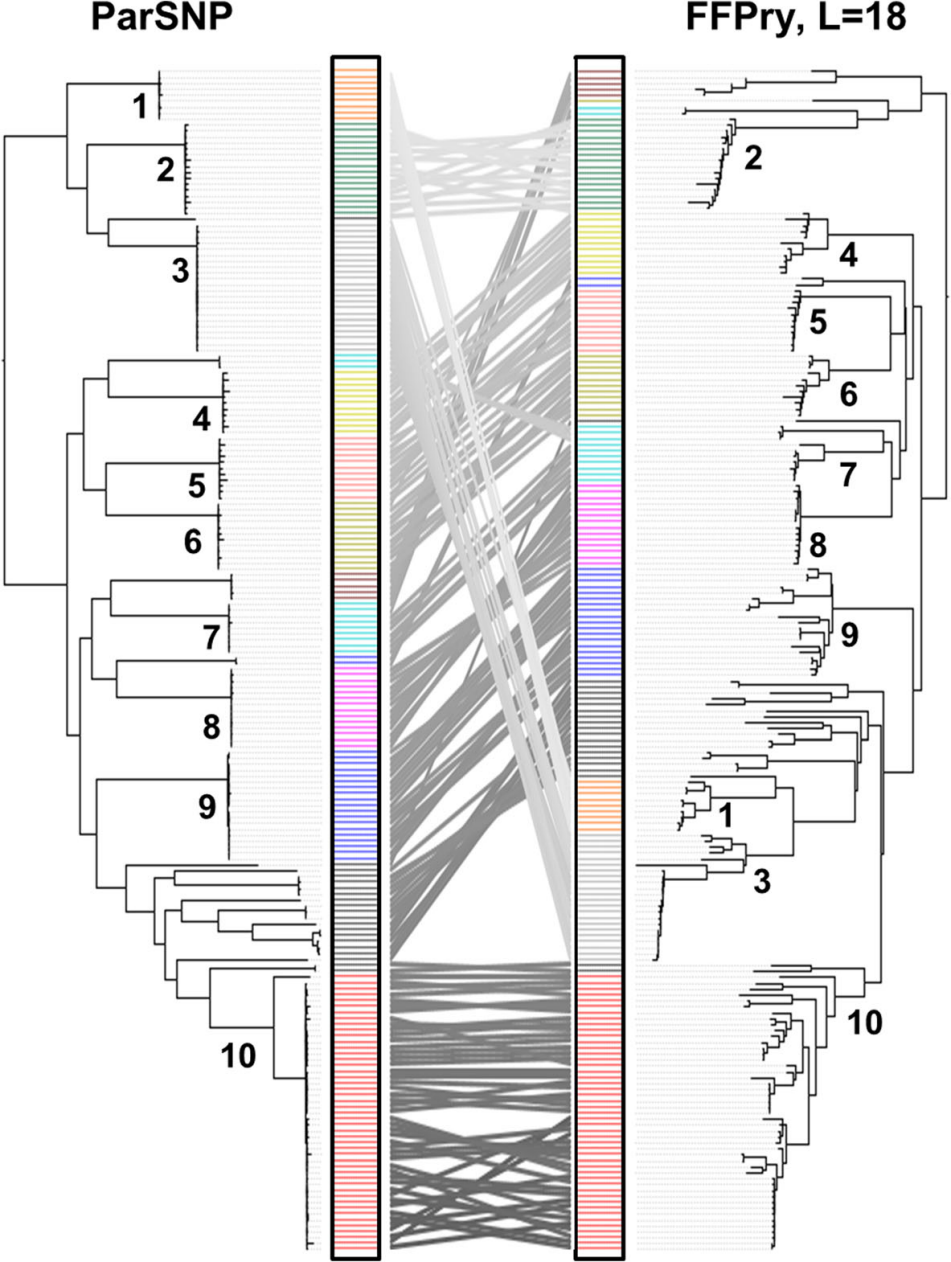
Comparison of *Salmonella enterica* phylogenetic trees based on core genome single nucleotide polymorphisms using ParSNP [27] and the alignment-free whole genome comparison with feature frequency profiling of purine-pyrimidine words (FFPry) with a word length (L) of 18 [28], using the 200 *Salmonella* genomes included here, visualised using the serovars (colored bar) and a tanglegram in the middle to indicate position of individual genomes in both phylogenetic trees. The left panel represents the phylogenetic tree based on core genome SNPs, while the right panel shows the phylogenetic tree obtained FFPry. Note that although the order of serotypes differs, isolates cluster generally according to serotype in both analysis methods, with the overall topology being similar. Clusters containing major serovars are indicated by numbers: 1. Johannesburg; 2. Muenster; 3. Schwarzengrund; 4. Worthington; 5. Altona; 6. Mbandaka; 7. Senftenberg; 8. Rissen; 9. Derby; 10. Typimurium and 4,[5],12:i:-

**Fig. 2:**
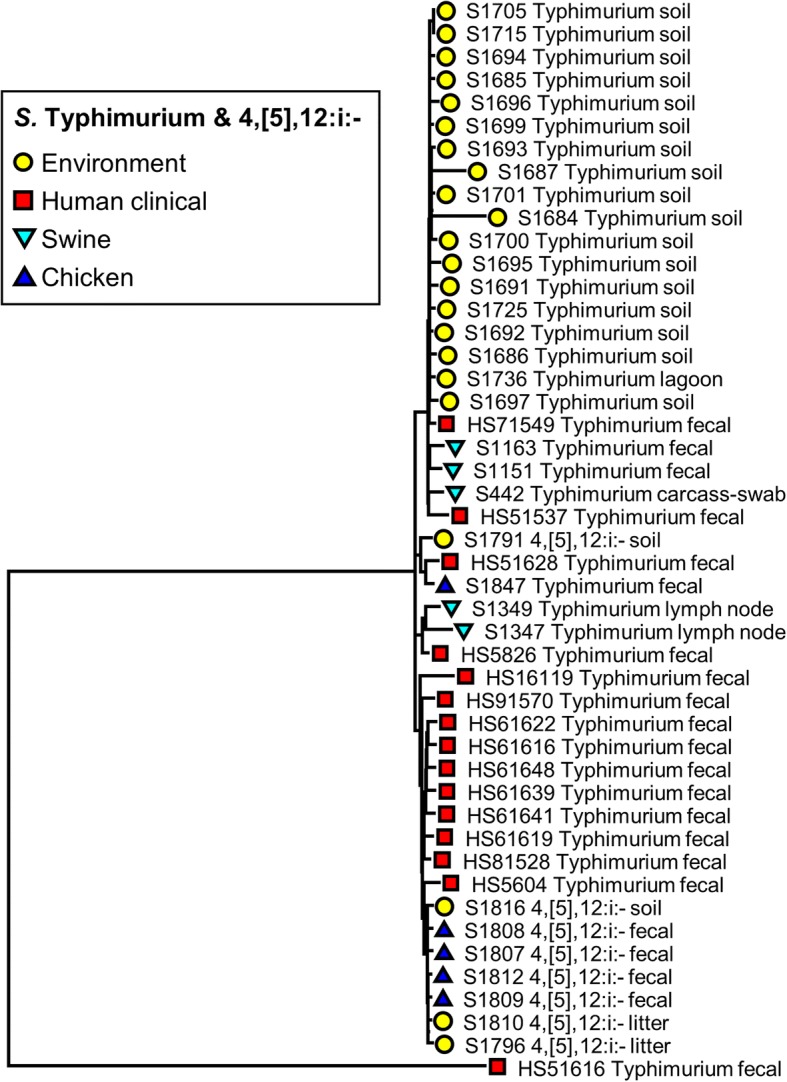
Phylogenetic tree of *S*. Typhimurium and *S*. 4,[5],12:i:- isolates (*n* = 47) recovered from human, swine, chicken, and environmental sources constructed using parSNP analysis. Colored markers indicate the source of each isolate, with more details added to the name of each isolate

**Fig. 3:**
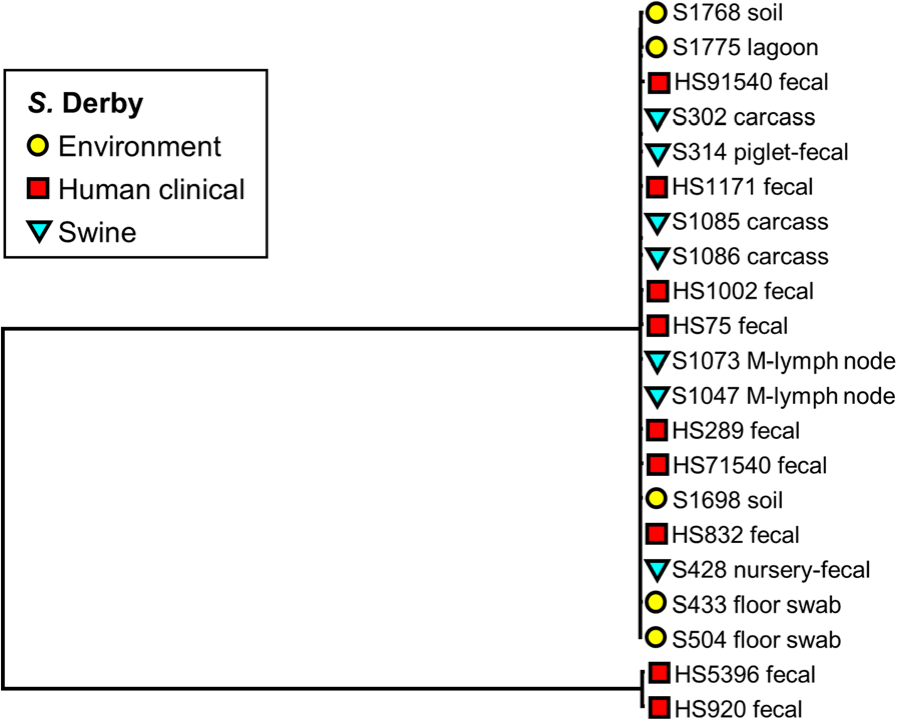
Phylogenetic tree of *S*. Derby isolates (*n* = 21) recovered from recovered from human, swine, and environmental sources constructed using parSNP analysis. Colored markers indicate the source of each isolate, with more details added to the name of each isolate

**Fig. 4:**
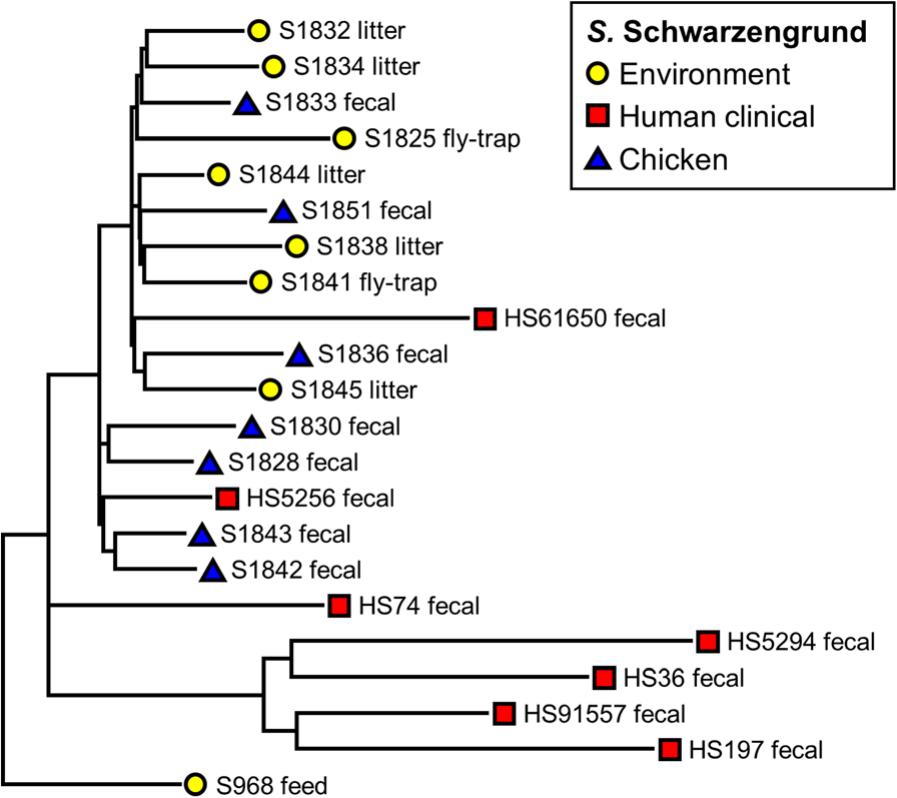
Phylogenetic tree of *S.* Schwarzengrund isolates (*n* = 22) recovered from recovered from human, chicken, and environmental sources constructed using parSNP analysis. Colored markers indicate the source of each isolate, with more details added to the name of each isolate

**Fig. 5:**
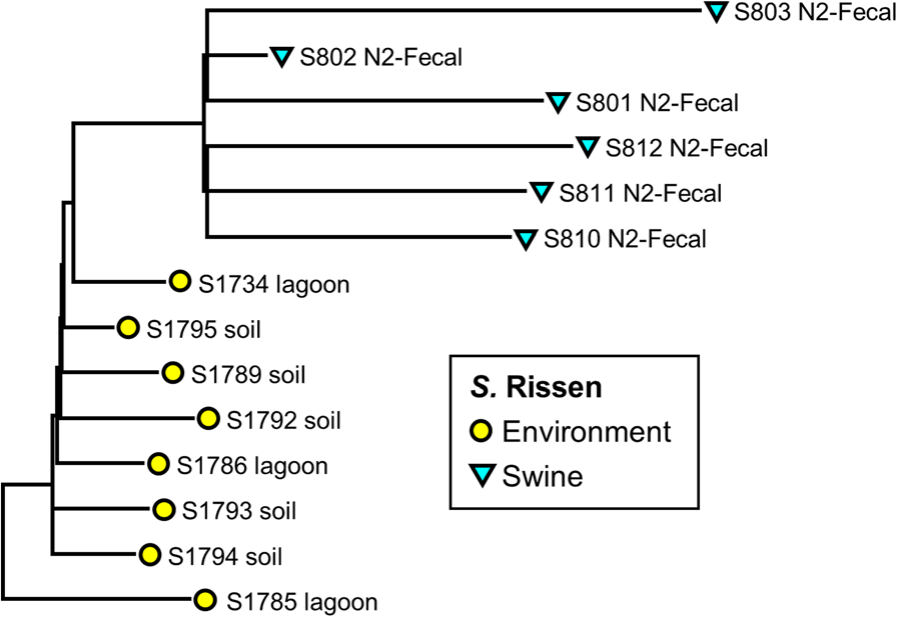
Phylogenetic tree of *S*. Rissen isolates (*n* = 14) recovered from recovered from swine and environmental sources constructed using parSNP analysis. Colored markers indicate the source of each isolate, with more details added to the name of each isolate

The 200 *Salmonella enterica* genomes were assessed for their phylogenetic relationships using core genome SNPs with the ParSNP program [27] and feature frequency profiling with the FFPry program [28]. Isolates clustered according to serotype with both analysis methods, and the topology of the resulting phylogenetic trees was very similar (Fig. 1). Although the order of specific serovars did differ, the 200 *Salmonella* genomes clustered into 10 different major groups matching the respective serovars in both parSNP and FFPry trees. The differences in order of the clusters between the FFPry and parSNP trees may be explained by the parSNP tree being based on the core genome only, thus excluding phages, plasmids and regions of horizontal gene transfer. In addition, many major serovar clusters were comprised of the genomes from different sources of origin including human, animal, and the environment. There were several singleton genomes that did not cluster into any major serotype-associated group. Therefore, these differences have a relatively small effect on the general structure of the trees and the clustering observed. ParSNP reported coverage over the genome for each run, and when all 200 *Salmonella* genomes were included, the average coverage was 77.6%. For the individual serovars, these were 89.4% (Fig. 2, Typhimurium and 4,[5],12:i:-), 85.3% (Fig. 3, Derby), 89.0% (Fig. 4, Schwarzengrund) and 97.9% (Fig. 5, Rissen).

We focused on these major clusters for serovars Typhimurium, Derby, Schwarzengrund, and Rissen (Figs. 2, 3, 4, 5). These clusters were comprised of the genomes from multiple sources. *S*. Typhimurium and *S*. 4,[5],12:i:- genomes recovered from human, swine, poultry, and environmental sources clustered together (Fig. 2). The genomes from the same origin have a close relationship as indicated by the positioning on the phylogenetic SNP tree. However, a human clinical fecal (HS71549) was closely grouped along with environmental isolates from the commercial swine farms. Another human case genome (HS5826) was placed near the swine samples on the tree. The genomes of serotype 4,[5],12:i:- recovered from both chicken fecal and environment were grouped close to each other, most likely because they originated from the same farm in Tennessee.

The isolates with serotype Derby showed little variation in the core genome, nor was any specific clustering linked with human, swine, and environmental sources (Fig. 3). In contrast, the isolates of *S.* Schwarzengrund (Fig. 4) showed isolation source-specific clustering of human isolates separate from the group of chicken fecal and environmental genomes, with the exception of two isolates from human clinical cases (HS5256 and HS61650). The environmental samples of this serotype were from the litter and the fly traps collected from the chicken farms. The genomes of *S*. Rissen clustered based on the source of isolates (Fig. 5). The swine fecal genomes were grouped together, while the soil and lagoon genomes even collected from the different farms and time points still clustered together and separated from swine branch.

### Detection of AMR genes, plasmid replicons, and virulence genes using WGS

**Fig. 6:**
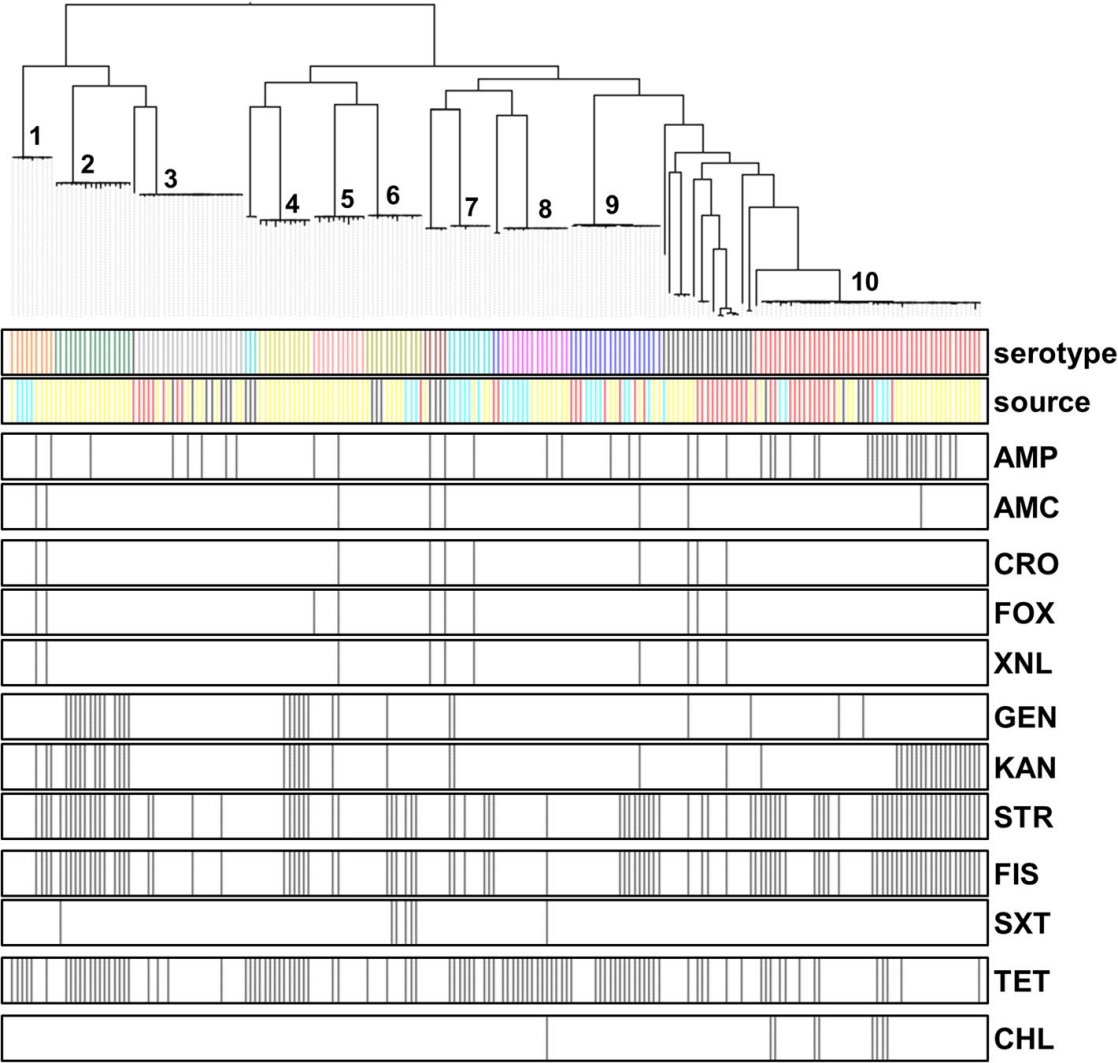
Distribution of phenotypic antimicrobial resistance in the genomes of the 200 *S. enterica* isolates included in this study. The isolates were clustered based on core genome SNPs using ParSNP, and the antimicrobial resistances are shown by black lines in the respective bars below. Antimicrobials used are grouped according to their class and mechanism: penicillins (AMP, ampicillin; AMC, amoxicillin and clavulanate [augmentin, AUG]); cephalosporins (CRO, ceftriaxone (AXO); FOX, cefoxitin; XNL, ceftiofur); aminoglycosides (GEN, gentamicin; KAN, kanamycin; STR, streptomycin); sulfanomides/folate inhibitors (FIS, sulfisoxazole; SXT, trimetroprim and sulfamethoxazole); tetracycline (TET) and chloramphenicol (CHL). For source, these were subdivided into four major classes: environment (yellow), human clinical (red), chicken (dark blue) and swine (light blue). Major serovars are indicated by numbers: 1. Johannesburg; 2. Muenster; 3. Schwarzengrund; 4. Worthington; 5. Altona; 6. Mbandaka; 7. Senftenberg; 8. Rissen; 9. Derby; 10. Typimurium and 4,[5],12:i:-. Full details of source can be found in Additional file 2: Table S1

The WGS data was used to detect the presence and absence of AMR genes, plasmid replicon, and virulence genes in the 200 *Salmonella* genomes (Figs. 6 and Additional file 1: Figure S1). Overall, the most common resistance genes detected were *sul*1 (32.5%), *tet*R (28.5%), and *tet*A (24%) (Additional file 2: Table S2). The three most frequent replicons, including ColRNAI, IncFIB, and IncFII were detected in 43%, 16%, and 15.5% of all *Salmonella* sequences, respectively (Additional file 2: Table S2). In addition, the 200 *Salmonella* genomes were also screened for virulence genes. One hundred and seventy-five virulence genes were detected in this study using WGS (Additional file 2: Table S2). All 200 isolates were positive for thirty-nine virulence genes, including *inv*A, *sip*B, *prg*H, *spa*, *org*A, *iro*N, *sif*A, and *sop*B (Additional file 2: Table S2).

### AMR correlation based on phenotypic (MIC) and genotypic data (WGS)

Genome sequence data were correlated with the phenotypic AMR profiles to evaluate the ability of WGS to predict phenotypic resistance (Figs. 6 and Additional file 1: Figure S1). The most frequent AMR phenotypes were resistance against streptomycin (STR; 57.5%), tetracycline (TET; 51%), and sulfisoxazole (FIS; 46%) (Additional file 2: Table S1). Resistance to azithromycin, ciprofloxacin and nalidixic acid was not detected in this study and, therefore, not included for evaluation. Overall, phenotypic resistance correlated strongly with the presence of corresponding AMR determinants using WGS (Table 2). The overall sensitivity of AMR coding genes present for predicting resistance across all antimicrobials was 87.61%, the specificity was 97.13%, the positive predictive value (PPV) was 88.35%, and the negative predictive value (NPV) was 96.93% as shown in Table 2. The genotypic prediction of phenotypic resistance to sulfisoxazole (FIS), tetracyclines (TET), and cephems (ceftriaxone, CRO; cefoxitin, FOX; ceftiofur, XNL) had a sensitivity over 90%, while the other sensitivity values for other antimicrobials was lower than 90%. The genotype prediction of phenotypic resistance to all antimicrobials, other than streptomycin (STR), had specificity greater than 91% (Table 2).

**Table 2:** Comparison between genotypic AMR prediction by WGS and phenotypic expression based on MIC levels of AMR *Salmonella* isolates (*n* = 200)

Antimicrobials^a^	Resistant by phenotype		Susceptible by phenotype		Sensitivity (%)	Specificity (%)	PPV^b^ (%)	NPV^c^ (%)
	WGS: AMR gene +	WGS: AMR gene -	WGS: AMR gene +	WGS: AMR gene -				
AMP	36	7	4	153	83.72	97.45	90	95.63
AMC	8	2	4	186	80	97.89	66.67	98.94
CRO	9	1	3	187	90	98.42	75	99.47
FOX	6	1	3	187	90	98.42	75	99.47
XNL	6	1	3	187	90	98.42	75	99.47
GEN	24	4	5	167	85.71	97.09	82.76	97.66
KAN	37	9	9	145	80.43	94.16	80.43	94.08
STR	98	17	10	75	85.22	88.24	90.74	81.52
FIS	87	5	4	104	94.57	96.29	95.6	95.41
SXT	6	1	1	192	85.71	99.48	85.71	99.48
TET	93	9	8	90	91.18	91.84	94.9	88.24
CHL	7	2	1	190	77.78	99.48	87.5	98.96
Overall					87.61	97.13	88.35	96.93

^a^ ampicillin (AMP), amoxicillin/clavulanic acid (AMC), ceftriaxone (CRO), cefoxitin (FOX), Ceftiofur (XNL), gentamicin (GEN), kanamycin (KAN), streptomycin (STR), sulfisoxazole (FIS), trimethoprim/sulfamethoxazole (SXT), and tetracycline (TET), chloramphenicol (CHL)

^b^ positive predictive value (PPV)

^c^ negative predictive value (NPV)

### Association of AMR genes, plasmid replicons, and virulence genes with different *Salmonella* serotypes using WGS

Serotypes were found to vary with regard to the presence/absence of AMR coding gene, plasmid replicon, and virulence gene using WGS approach based on the odds ratio to evaluate their associations (Table 3). Significant (*P* < 0.05) associations between *S*. Typhimurium and *S*. 4,[5],12:i:- with AMR genes were observed, including *aad*A25, *sul*1, *tet*A, and *tet*G, while the *aad*A1, *aad*A2, *tet*A, and *tet*R genes were found significantly associated with *S*. Derby (Table 3). On the other hand, AMR genes including *aph*(3″)-Ib, *aph*(6)-Id, *str*A, and *str*B were significantly detected in *S*. Schwarzengrund (Table 3). Several significant (*P* < 0.05) associations between plasmids and *Salmonella* serotypes were also observed, including IncFIB and IncFII in serotypes Typhimurium, 4,[5],12:i:-, and Schwarzengrund, while IncQ2 was significantly found in serotype Derby.

**Table 3:** AMR determinant, plasmid replicon, and virulence gene detections based on WGS in *Salmonella* serotypes

Characteristic:	*S*. Typhimurium & *S*. 4,[5],12:i:-	*S*. Derby	*S*. Schwarzengrund
AMR gene (OR)^a^	*aad*A25 (11.05) *sul*1 (2.18) *tet*A (0.23) *tet*G (∞)	*aad*A1 (4.22) *aad*A2 (3.2) *tet*A (4.57) *tet*R (10.44)	*aph*(3″)-Ib (48.9) *aph*(6)-Id (25.25) *str*A (48.9) *str*B (40.48)
Plasmid (OR)^a^	colRNAI (5.07) IncFIB (8.4) IncFII (10.51)	IncQ2 (∞)	IncFIB (7.22) IncFIC (∞) IncFII (20.53)
Virulence gene (OR)^a^	*pef*A (224) *spv*B (268.24) *ssp*H1 (3.28)	*gtr*A (5.38) *sse*J/K1/L (∞) *sse*K2 (4.5)	*cdt*B (∞) *iuc*A/B/C/D (92.27) *iut*A (92.27) *spv*B (0)

^a^ An odds ratio (OR) of 0 indicates the absence of that gene in a given *Salmonella* serotype, while the OR of infinity (∞) indicates that the mentioned gene was detected only in a specific serotype and none of the other serotypes

Only the odds ratios with *P*-value < 0.05 are shown

As highlighted previously, several major virulence genes were detected in all *Salmonella* isolates in our study (Additional file 2: Table S2). However, *pef*A, *spv*B, and *ssp*H1 were specifically detected in serovar Typhimurium and 4,[5],12:i:- (Table 3). *S.* Schwarzengrund genomes were significantly associated with the presence of *cdt*B, *iuc*, and *iut*A genes, while *gtr*A and *sse* genes were significantly detected in *S*. Derby (Table 3).

## Discussion

The objective of this study was to characterize *Salmonella* serovar, AMR determinants and virulence genes using whole genome sequencing. The 200 *Salmonella enterica* genomes were isolated from different sources of origin including human, swine, poultry, and environment, and were analyzed using the core genome SNP-based analysis and the alignment-free analysis method FFP. The phylogenetic trees obtained from parSNP and FFPry showed that the clusters observed matched *Salmonella* serovars (Fig. 1). The branch length in FFP-based trees is more representative of differences over the whole genome, which may be due to differential plasmid, prophage content, or other accessory genome [28], while SNP-based trees use the core genome derived from whole-genome alignment and read mapping for phylogeny construction [27, 28]. The major difference of the SNP- and FFP-based analyses was in the order of the serovar clusters within the tree, however, the overall approach selected had relatively little effect on the topology of the phylogenetic trees (Fig. 1). A number of studies have reported the use of SNP-based analysis as a potential molecular subtyping tool for outbreak investigation in multiple *Salmonella* serovars including Dublin [29], Enteritidis [22, 30], Heidelberg [31], Manhattan [21], Montevideo [32, 33], and Typhimurium [34–36].

The phylogenetic analysis based on WGS-derived SNPs has been shown to provide greater cluster resolution than the gold standard subtyping method, pulsed-field gel electrophoresis (PFGE), resulting in discrimination of outbreak-related human clinical isolates and food or environmental origins [21–23]. ParSNP was run for all the 200 *Salmonella* genomes and separately for each of the serovars. Although the separate comparisons indeed increased the core genome component as would be predicted, this was not a major increase, and we do not expect that the removal of the outliers will have a significant effect on the trees. In concordance with those prior literatures, the current study found that the core genome SNP-based trees of individual *Salmonella* serovar including Typhimurium and 4,[5],12:i:- (Fig. 2), Schwarzengrund (Fig. 4), and Rissen (Fig. 5) were mostly clustered based on source of origin. However, there were some exceptions in each individual tree. As shown in Fig. 2, some clinical *S.* Typhimurium isolates (HS71549, HS51537, and HS51628) were closely related to the environmental, swine, and chicken isolates, respectively. The Schwarzengrund cluster in Fig. 4 showed that the *Salmonella* isolates from chicken feces clustered with the isolates obtained from environmental isolates which were derived from the same farm. There was no isolation source-dependent clustering in *S.* Derby (Fig. 3), with genomes from human, swine and environmental isolates clustering together. These findings can point towards the potential transmission of *Salmonella* among humans, animals and the environment and support the idea of zoonotic transmission, while independent human sub-clustering in each serovar might be referred to human-to-human transmission. However, the human *Salmonella* isolates included in our analysis were only from the North Carolina State Public Health Laboratory which might not be represent all the human clinical cases. According to the same timeline as human *Salmonella* outbreaks belonged to the independent human sub-clusters, the animal/environmental sources which may have a chance to group with those human sub-clusters were not scheduled for sampling. In addition, *S.* 4,[5],12:i:- has been defined as a monophasic variant of serovar Typhimurium because of their antigenic and genetic similarities, and the characterization of *S.* 4,[5],12:i:- using the typical molecular approaches revealed that *S*. Typhimurium is the direct ancestor of *S.* 4,[5],12:i:- [37]. Even the two serovars were clustered together (Fig. 2), parSNP-based subtyping could be a suitable analysis applied to differentiate these serovars. In contrast to *S*. Derby (Fig. 3), the sources of origin cannot be differentiated using parSNP analysis. This serovar has a highly homogeneous genetic composition and can be carried by different hosts [38]. Moreover, the MLST database (http://mlst.ucc.ie/mlst/mlst/dbs/Senterica/) indicates that Derby is a polyphyletic serovar, having originated from more than one common ancestor, and possesses several distantly related sequence types (ST) [39]. Thus parSNP-based analysis might not be an appropriate method for this serovar. However, a recent study in China reported that the clustered regularly interspaced short palindromic repeats (CRISPRs) could be a useful subtyping tool for *S*. Derby in molecular epidemiological investigations [40]. Though the SNP typing is the reliable tool for genomic and epidemiologic studies, it is not without limitations. SNP-based analysis requires alignment of whole genome sequences and only utilizes the core genome, which may be less sensitive as a result. In addition, this method is still limited to the intragenus analysis of closely related species and strains [27, 41].

The FFP phylogenetic clustering is an effective tool that relies on an alignment-free approach for genomic evolution study. Unlike parSNP-based method which focuses on core genome, the phylogenetic trees acquired from FFP-based method are affected by recombinant genes and/or horizontal gene transfer including plasmid, prophage, and other accessory gene contents [28]. However, the main clustering structure is not significantly different (Fig. 1). The advantages of FFP-based analysis are that it is independent of a reference genome, and has lower hardware requirements. Additionally, FFP analysis can be performed with whole genome shotgun samples as it is not affected by contig orientation, and contig order [28]. FFP-based analyses has been reported in a number of bacterial genomic studies, including *Helicobacter pylori* [28], *Bacillus* spp*.* [42], *Escherichia coli* [43, 44], and *Shigella* [44]*.* These studies have revealed that the FFP method can contribute to the phylogenetic clusters based on geographic relation and outbreak detection, and could provide a complementary analysis approach. Our study is the first to utilize the alignment-free FFP analysis in *Salmonella* and compare it to the core genome SNP-based analysis. We found that the phylogenetic clusters from these methods were similar in term of serovar characterization, but the branching varied due to differences in analysis approaches (Fig. 1). While SNP- and MLST-based methods are likely to continue to be the default choice for subtyping and comparative genomics in *Salmonella*, the FFP method can serve as a useful alternative method requiring relatively low-powered computing resources [28].

Antimicrobials are reported extensively used in food animal production to treat clinical disease, to prevent and control common diseases, and to enhance animal growth [45]. Tetracycline and tylosin are frequently mixed in animal feed for disease prevention and growth promotion purposes in commercial swine and poultry systems [45, 46]. In accordance to the high percentages of phenotypic tetracycline and sulfisoxazole resistance were reported in our result. The WGS revealed a number of tetracycline and sulfisoxazole resistance genes such as *tetA*, *tetB*, *tetC*, *tetR*, *sul1*, and *sul2* (Additional file 2: Table S2). Of interest, gene mechanisms of tetracycline resistance including the efflux genes, the ribosomal protection and enzymatic genes were suggesting a possible ecological role for specific wide spread of tetracycline resistance [47]. However, AMR genes especially tetracycline and sulfonamide were also detected in livestock production surrounding even when antimicrobial drugs were not administered to animals [47, 48].

In this study, we have shown that WGS is an excellent tool for accurately predicting antimicrobial resistant phenotype in human, animal, and environment associated multiple *Salmonella* serovars, as WGS predictions and phenotypic resistance matched well with high sensitivity and specificity in our study. Overall, the resulting resistance genotypes correlated with 87.61% sensitivity and 97.13% specificity to the resistance phenotype (Table 2). Among the discordant results in our study, the lowest specificity of AMR prediction was observed for streptomycin which accounted for the presence of streptomycin-resistance genes but lacked phenotypic resistance. This finding was in concordance with the previous studies in *Salmonella* [24, 49] and *E. coli* [25, 50]. The streptomycin discrepancies have been commonly detected in other studies too because streptomycin is not used to treat enteric infections, and as such, results in the absence of precise clinical breakpoint for streptomycin susceptibility in *Salmonella* and *E. coli* [24]. Although the *strA*/*strB* and *aadA* genes were detected, the *strA*/*strB* genes conferred higher resistance than *aadA* genes [25, 51]. Thus, the presence of *aadA* genes by in silico method may not result in streptomycin resistance phenotypically. In addition, the mechanism of streptomycin resistance is frequently due to lacking of the gene expression as well as mutations in the 16S rRNA gene leading to difficulty of phenotypic prediction [50, 52]. Our results suggest that the refinement of WGS-based AMR prediction could be beneficial and can definitely enhance the monitoring of AMR strains and determinants detected in humans, foods, animals, and environment.

The *Salmonella* serovars significantly correlated with the presence/absence of AMR genes, plasmid replicons, and virulence genes. We observed specific AMR genes in each *Salmonella* serovar (Table 3). This result along with the phylogenetic relatedness revealed that the type of serovar in discussion had the greatest impact on AMR characterization. Previous studies reported the presence of AMR genes has been shown to be primarily associated with a particular host and is not frequently transmitted among different species which in accordance to our finding (Additional file 2: Table S2) [20, 49]. Multiple plasmid replicons were detected in this study using WGS method (Additional file 2: Table S2). Plasmids were observed specific to *Salmonella* serovar that was very similar to the AMR genes (Table 3). This is in accordance to our previous study that the plasmid profiles were correlated to *Salmonella* serovar and incompatibility (Inc) groups [13]. The IncF (both FI and FII) family found across the different *Salmonella* serovars in our study is known to be a well-adapted and commonly distributed plasmid among members of the *Enterobacterceae* family [53, 54]. Although our data cannot fully explain the transmission of AMR determinants among various species, they are in line with previous studies that reported on the role of animals and environment as important sources of multiple AMR genes as well as plasmids, and that transmission can occur by horizontal gene transfer [13, 55, 56].

Multiple virulence genes were identified among the several *Salmonella* serovars across different sources by WGS (Additional file 2: Table S2). These genes have been described to be involved in several processes important for *Salmonella* transmission and infection, including adhesion, type III secretion system (T3SS), host recognition/invasion, filamentous formation, magnesium uptake, iron acquisition, and regulation of stress factors. Our data showed that *Salmonella* isolates recovered from animal or/and environmental sources contained the same virulence genes as carried by human clinical isolates. Along with the phylogenetic analysis, these findings support our view that the high frequency of virulence genes detected in food animal and environment may be transmitted and cause infections in humans, a suggestion that has been previously made in prior studies [57–59]. Figueira et al. (2013) reported that the lack of *sseJ*, a particular virulence gene makes *S.* Typhimurium strain became more heterogenous [60]. In our study, this gene was only present in *S*. Derby (Table 3) which may relate to the non-source-dependent clustering found in this homogenous serovar as mentioned previously (Fig. 3). One of the typhoid-associated virulence factors, the cytolethal distending toxin *cdtB*, was detected in all isolates of Schwarzengrund, Johannesburg, and Muenster (Additional file 2: Table S2), which was similar to a previous study that detected this gene in *S.* Schwarzengrund [61]. The *cdtB* encodes the typhoid toxins of *S.* Typhi and is not reported from a wider distribution among non-typhoidal *Salmonella* serovars (NTS) [57, 61]. However, there were a few reports of the prevalence of this virulence gene in several NTS, including Javiana [57], Montevideo, Schwarzengrund, and Bredeney [61]. This data suggested that the *cdtB* toxin may contribute to the pathogenicity in human and animal.

## Conclusions

WGS is a helpful tool to assess the phylogenetic relations among multiple serotypes, AMR and virulenuce gene evaluation and assist in the molecular epidemiological studies of foodborne pathogens. The SNP-based and FFP-based analysis provided the higher resolution *Salmonella* phylogenetic trees that could differentiate the isolates recovered from human, animal, and environment. In addition, WGS is a useful tool for AMR prediction, plasmid replicon, and virulence gene detections. Our study shows the close relationship between *Salmonella* isolates associated with different hosts, which is supportive of possible zoonotic transmission. This is seen among multiple serotypes, and the prevalence of AMR genes, plasmid replicons and virulence genes that were identical in different species and could potentially highlight exchange of serovars across different hosts.

## Methods

### *Salmonella* isolates selection

The 200 *Salmonella* isolates included are from multiple serovars collected from multiple sources, including human, swine, poultry, and agricultural environment, and used for WGS (Table 1). The serovars were selected across multiple time points between the years 2009–2016. The human *Salmonella* isolates were from stool samples from clinical cases received from the North Carolina State Public Health Laboratory (*n* = 44). Swine isolates (*n* = 32) originated from fecal, lymph nodes, and carcass swab samples from commercial farms in North Carolina, while poultry isolates (*n* = 22) were from chicken fecal samples collected from sustainable farms in North Carolina and Tennessee. Environmental isolates (*n* = 102) were collected from commercial farms and sustainable farms in NC and TN. The list of isolates and details were tabulated in Additional file 2: Table S1. All samples were stored in Brucella broth at − 80 °C until further characterization.

### Phenotypic antimicrobial resistance testing

The antimicrobial susceptibility and the minimum inhibitory concentration (MIC) profile of each *Salmonella* isolate was determined by the broth microdilution method using the gram-negative Sensititre™ (CMV3AGNF) plate (Trek Diagnostic Systems, OH) in accordance with the guidelines and interpretations published by the Clinical and Laboratory Standards Institute (CLSI) [62, 63] and National Antimicrobial Resistance Monitoring System (NARMS) [64]. The panel of 15 antimicrobials tested include amoxicillin/clavulanic acid (AMC, suppliers abbreviation AUG; 1/0.5–32/16 μg/ml; breakpoint > 32/16), ampicillin (AMP; 1–32 μg/ml; breakpoint > 32), azithromycin (AZI; 0.12–16 μg/ml; breakpoint ≥32), cefoxitin (FOX; 0.5–32 μg/ml; breakpoint ≥32), ceftiofur (XNL; 0.12–8 μg/ml; breakpoint ≥8), ceftriaxone (CRO, suppliers abbreviation AXO; 0.25–64 μg/ml; breakpoint ≥4), chloramphenicol (CHL; 2–32 μg/ml; breakpoint ≥32), ciprofloxacin (CIP; 0.015–4 μg/ml; breakpoint ≥4), gentamicin (GEN; 0.25–16 μg/ml; breakpoint ≥16), kanamycin (KAN; 8–64 μg/ml; breakpoint ≥64), nalidixic acid (NAL; 0.5–32 μg/ml; breakpoint ≥32), streptomycin (STR; 2–64 μg/ml; breakpoint ≥32), sulfisoxazole (FIS; 16–256 μg/ml; breakpoint ≥256), trimetroprim/sulfamethoxazole (SXT; 0.12/2.38–4/76 μg/ml; breakpoint ≥4/76), and tetracycline (TET; 4–32 μg/ml; breakpoint ≥16). *E. coli* ATCC25922 was used as internal quality control. The *Salmonella* isolates with MICs in the intermediate level were categorized into susceptible to avoid overestimation of resistance.

### Genome library preparation and sequence assembly

The *Salmonella* isolates (*n* = 200) were cultured overnight at 37 °C on Luria-Bertani (LB) agar. Genomic DNA were extracted using DNeasy blood and tissue kit (Qiagen, CA). DNA concentrations were quantitated using the Qubit 4.0 Fluorometer for double-strand-DNA high-sensitivity assay kit (Thermo Fisher Scientific, MA). Genomic libraries were prepared using the Nextera XT kit (Illumina, CA) for multiplexed sequencing. WGS were performed on the Illumina MiSeq platform with 2*250 bp paired-end (PE) reads (MiSeq reagent kit, version 3). Genomes were assembled using SPAdes 3.10.1 [65], with contigs < 200 bp and coverage < 10-fold excluded from downstream analyses. The assemblies were checked for quality parameters (genome size, largest contig, N50 and L50 values) using QUAST v. 4.5 [66].

### *Salmonella* serotyping and *Salmonella* in silico typing resource (SISTR)

The animal and environmental *Salmonella* isolates were initially sent to the National Veterinary Services Laboratories (NVSL) at Ames, Iowa for serotyping using the Kauffman-White scheme, while the human serotyping was conducted at the North Carolina State Public Health Laboratory. All 200 *Salmonella* genomes were analyzed using the *Salmonella* in silico Typing Resource (SISTR) software (https://lfz.corefacility.ca/sistr-app/) for serovar prediction. The SISTR module utilizes O (somatic) antigen, H (flagellar: H1 and H2) antigen, and/or serogroup-specific probes particularly designed for *Salmonella* Genoserotyping Array (SGSA) [26]. The results from SISTR interpretation were compared to the traditional Kauffman-White serotyping. The serovar prediction was confirmed by phylogenetic analysis using core genome parSNPs and FFP analysis as described below.

### Alignment-free feature frequency profiling and core genome SNPs analysis

The 200 *Salmonella* genomes were identified for core genome SNPs and were clustered using the ParSNP program from the Harvest suite [27], using the “-a 13” and “-x” settings [28], which respectively invoke a smaller (a)NCHOR window for higher resolution mapping [27], and the PhiPack module, which excludes SNPs located in regions of recombination. For the parSNP tree shown in Fig. 1, a random genome was selected from the 200 genomes using the “-r!” switch. In addition, trees were generated using a single representative of 11 serovars, which did not result in noticeable differences in tree topology (data not shown). An alignment-free feature frequency profiling using purine-pyrimidine words (FFPry) was performed with the FFP version 3.19 suite of programs (http://sourceforge.net/projects/ffp-phylogeny/) [67, 68], utilizing the FFPry generated phylogenetic tree [28]. Treegraph v2 [69] and Figtree (http://tree.bio.ed.ac.uk/software/figtree/) were used to annotate and visualize the phylogenetic trees.

### Determination of *Salmonella* virulence, plasmid replicons, and antimicrobial susceptibility determinants

The genotyping by in silico method for 200 *Salmonella* sequences were done by annotating assembled genomes via Prokka v1.12 [70]. The contigs were submitted to PlasmidFinder [71], and ResFinder [72] modules to determine the existing plasmid replicon types, and AMR genes, respectively. Virulence genes were identified with an in-house workflow using SRST2 v0.1.4.5 [73]. The Illumina raw reads were mapped against chromosomal and plasmid virulence genes found in the Virulence Factor Database for *Salmonella* (VFDB) which currently contains 2017 genes database associated with virulence in *Salmonella* [http://www.mgc.ac.cn/VFs/status.htm] [74]. Finally, the presence/absence of AMR determinants, plasmid replicons, and virulence genes were calculated for association with *Salmonella* serotype using odds ratios along with Chi-square test or Fisher’s exact test with the *P*-value level < 0.05 of significance. All statistical analysis was carried out using R version 3.1.2 (R foundation for statistical computing, Vienna, Austria).

### Correlation of susceptibility phenotypes and genotypes

All phenotypic characters were generated from the 200 *Salmonella* isolates by broth microdilution (Sensititre™) antimicrobial susceptibility testing previously described. Each interpretation of resistant or susceptible to a given antimicrobial drug were compared to the presence or absence of known corresponding resistance genes and/or specific structural gene mutations detected by the WGS. The percentage of correlation between resistant phenotypes and genotypes were calculated. The phenotypic results were counted as the reference outcome, sensitivity was calculated by dividing the number of isolates that were genotypically resistant by the total number of isolates exhibiting clinical resistant phenotypes. Specificity was also calculated by dividing the number of isolates that were genotypically susceptible by the total number of isolates with susceptible phenotypes. The percentages of positive predictive values (PPV) and negative predictive values (NPV) were calculated as well.

### Accession numbers

Paired-end reads for the 200 *Salmonella* isolates in this study have been deposited in the National Center for Biotechnology Information (NCBI)’s under the Bioproject accession number PRJNA293224. Individual Sequence Read Archive (SRA) accession numbers have been tabulated in Additional file 2: Table S1.

## Additional files

Additional file 1: **Figure S1.** Distribution of phenotypic antimicrobial resistance of *Salmonella* isolates based on FFPry. (TIF 1823 kb)

Additional file 2: **Table S1.** *Salmonella* isolates that were sequenced and constructed for this research. **Table S2.** AMR, plasmid, and virulence genes. (XLSX 253 kb)

## Abbreviations

AMC, AUG: amoxicillin/clavulanic acid
AMP: Ampicillin
AMR: Antimicrobial resistance
AZI: Azithromycin
CHL: Chloramphenicol
CIP: Ciprofloxacin
CRISPR: Clustered regularly interspaced short palindromic repeats
CRO, AXO: Ceftriaxone
FFP: Feature frequency profiling
FIS: Sulfisoxazole
FOX: Cefoxitin
GEN: Gentamicin
KAN: Kanamycin
MCR: Plasmid-mediated colistin resistance
MDR: Multidrug resistant
MIC: Minimum inhibitory concentration
MLST: Multilocus sequence typing
MLVA: Multilocus variable-number tandem repeat analysis
NAL: Nalidixic acid
NPV: Negative predictive value
NTS: Non-typhoidal *Salmonella*
PFGE: Pulsed-field gel electrophoresis
PPV: Positive predictive value
SNP: Single nucleotide polymorphism
STR: Streptomycin
SXT: Trimetroprim/sulfamethoxazole
TET: Tetracycline
WGS: Whole genome sequencing
XNL: Ceftiofur
