# Supplementary figures and images for: Whole genome sequencing analysis of multiple Salmonella serovars provides insights into phylogenetic relatedness, antimicrobial resistance, and virulence markers across humans, food animals and agriculture environmental sources
Source: BMC Genomics. 2018 Nov 6;19:801. doi: 10.1186/s12864-018-5137-4 (PMC6218967; doi:10.1186/s12864-018-5137-4)

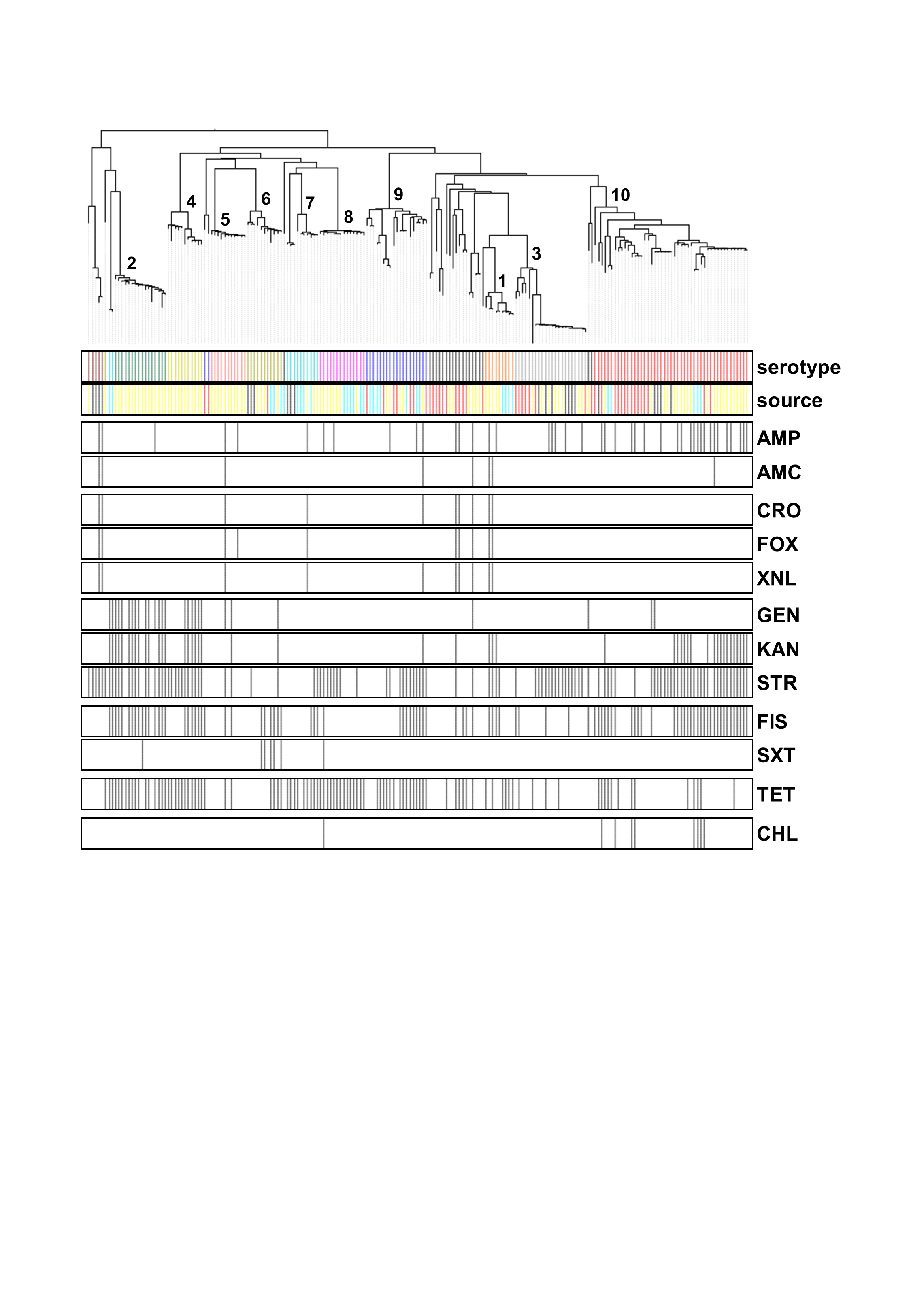

Supplement: Supplementary file 1 — Figure S1. Distribution of phenotypic antimicrobial resistance of Salmonella isolates based on FFPry. (TIF 1823 kb) [file 12864_2018_5137_MOESM1_ESM.tif]
